# Editorial: Advances in the neuromusculoskeletal modeling of injuries, diseases, and clinical treatments

**DOI:** 10.3389/fnins.2024.1463130

**Published:** 2024-08-08

**Authors:** Florian Michaud, Gil Serrancolí, Carlos Quental, Bernardo Innocenti

**Affiliations:** ^1^Laboratory of Mechanical Engineering, Campus Industrial de Ferrol, Universidade da Coruña, Ferrol, Spain; ^2^Department of Mechanical Engineering, Universitat Politecnica de Catalunya, Barcelona, Spain; ^3^IDMEC, Instituto Superior Técnico, Universidade de Lisboa, Lisbon, Portugal; ^4^BEAMS Department (Bio Electro and Mechanical Systems), École Polytechnique de Bruxelles, Université Libre de Bruxelles, Brussels, Belgium

**Keywords:** neuromusculoskeletal (NMS) model, musculoskeletal modeling, injury prevention, sport performance, disorder assessment, movement simulation, muscle force

Computer modeling and simulation of the human neuromusculoskeletal system have been employed to explore how the central nervous system manages movement and to assess the physical demands of various activities. The primary aim of these endeavors is to prevent injuries, diagnose pathologies, and evaluate and enhance treatments for movement impairments. These simulations offer valuable alternatives to *in vivo* experimental measurements of muscle forces, which are often impractical due to their invasiveness. Nevertheless, using computational tools presents several challenges: (1) capturing accurate movement efficiently and cost-effectively for clinical feasibility; (2) addressing the occasionally inconsistent correlation between muscle forces and EMG signals; and (3) personalizing muscle models to accurately represent both mechanical and neurological phenomena. Recent advances suggest that neuromusculoskeletal models can predict post-treatment function objectively and help identify treatment designs that optimize patient outcomes through numerical optimization.

Since Hill's early muscle models were introduced over 50 years ago, computer modeling and simulation of muscle activity have become a well-studied field that has greatly advanced injury prevention, pathology evaluation, treatment of movement impairments, and the design of assistive devices. The primary aim of this Research Topic is to offer a comprehensive collection of research and discussions focused on improving current methodologies and techniques for developing neuromusculoskeletal models related to diseases, injuries, diagnosis, rehabilitation, surgical interventions, and assistive aids.

The following paragraphs offer a concise overview of the content of each contribution included in the Research Topic.

In the first work (Michaud et al.), the authors present an innovative muscle fatigue model comprising four compartments. This model differentiates between the short-term fatigued state, associated with metabolic inhibition, and the long-term fatigued state, which simulates central fatigue and potential microtraumas. Through recent experimental measurements during both short- and long-duration exercises, they validated their approach and also demonstrated the limitations of the classic three-compartment model in handling any time-varying force profile.

In the second work (Zhu et al.), the study proposes an innovative and effective skeleton motion analysis method through the intricate integration of Transformer, Graph Neural Networks, and Generative Adversarial Networks. Significant experimental results have been achieved in the field of neuromusculoskeletal models. In-depth comparisons and analyses demonstrate the apparent superiority of the method in optimizing sports training and preventing injuries.

The third study (Shanbhag et al.) presents a musculoskeletal postural control model that incorporates complex sensor feedback from somatosensory, vestibular, and visual systems, accounting for realistic neural delays. This model successfully maintains balance in both unperturbed and perturbed conditions. It serves as a foundational tool for simulating and characterizing the movement behaviors of individuals with neurological disorders such as Parkinson's disease.

The case study presented in the fourth work (Li et al.) combined comprehensive walking data with personalized neuromusculoskeletal computer models to provide a thorough assessment of pre- to post-surgery changes in walking function (ground reactions, joint motions, and joint moments) and neural control (muscle synergies). This assessment was conducted for a single pelvic sarcoma patient who underwent internal hemipelvectomy surgery with custom prosthesis reconstruction. Their findings reveal significant alterations in the participant's post-surgery walking function and coordination of lower-limb muscles in both legs, as quantified through experimental data, despite minimal abnormalities being visually observed.

The aim of the fifth study (Noteboom et al.) was to compare musculoskeletal shoulder loads and potential injury risk during several bench press variations. The authors employed a musculoskeletal shoulder model to estimate joint reaction forces in the glenohumeral and acromioclavicular joints to observe the effects of bench press technique variations. The results of this study can contribute to safer bench press training guidelines.

In summary, the papers assembled in this Research Topic contribute either by proposing methods to optimize sports training and prevent injuries (Michaud et al.; Zhu et al.; Noteboom et al.), or by simulating and characterizing movement behaviors in individuals with neurological (Shanbhag et al.) or physical disorders (Li et al.) through neuromusculoskeletal modeling. This Research Topic highlights the diverse applications and advancements facilitated by neuromusculoskeletal modeling across various fields of research and clinical practice.

## Author contributions

FM: Writing – original draft, Writing – review & editing. GS: Writing – original draft, Writing – review & editing. CQ: Writing – original draft, Writing – review & editing. BI: Writing – original draft, Writing – review & editing.

